# Supplementary material for: A Global Screen for Assembly State Changes of the Mitotic Proteome by SEC-SWATH-MS
Source: Cell Syst. 2020 Feb 26;10(2):133–155.e6. doi: 10.1016/j.cels.2020.01.001 (PMC7042714; doi:10.1016/j.cels.2020.01.001)

O60341 | KDM1A_HUMAN | KDM1A AOF2 KDM1 KIAA0601 LSD1
Monomer MW [kDa]: 92.903 Monomer expected elution fraction: 41

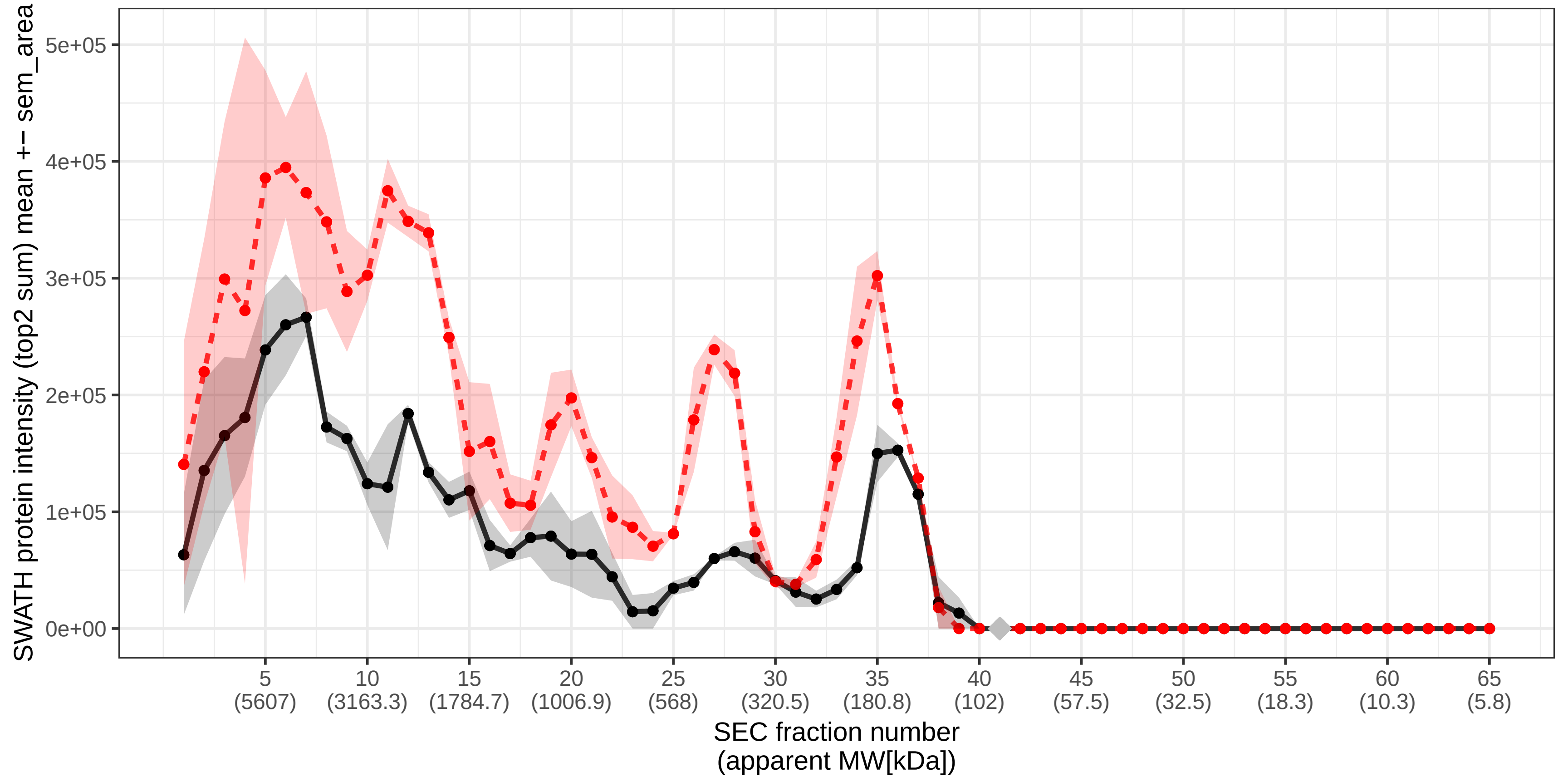

Supplement: Data S1. SEC-SWATH-MS Protein Chromatograms, Related to Figure 1 [file mmc6.zip › SECchrom_O60341_KDM1A_HUMAN_KDM1A_AOF2_KDM1_KIAA0601_LSD1.pdf]
